# Novice-performed point-of-care ultrasound for home-based imaging

**DOI:** 10.1038/s41598-022-24513-x

**Published:** 2022-11-28

**Authors:** Nicole M. Duggan, Nick Jowkar, Irene W. Y. Ma, Sara Schulwolf, Lauren A. Selame, Chanel E. Fischetti, Tina Kapur, Andrew J. Goldsmith

**Affiliations:** 1 Department of Emergency Medicine, Brigham and Women’s Hospital, 75 Francis Street, NH-2, Boston, MA 02115 USA; 2 Department of Radiology, Brigham and Women’s Hospital, Boston, USA; 3 Division of General Internal Medicine, University of Calgary, Calgary, Canada; 4 University of Connecticut School of Medicine, Farmington, USA

**Keywords:** Ultrasonography, Diagnosis, Disease prevention, Health services, Public health

## Abstract

Patient-performed point-of-care ultrasound (POCUS) may be feasible for use in home-based healthcare. We investigated whether novice users can obtain lung ultrasound (LUS) images via self-scanning with similar interpretability and quality as experts. Adult participants with no prior medical or POCUS training, who were capable of viewing PowerPoint slides in their home and who could hold a probe to their chest were recruited. After training, volunteers self-performed 8-zone LUS and saved images using a hand-held POCUS device in their own home. Each 8-zone LUS scan was repeated by POCUS experts. Clips were independently viewed and scored by POCUS experts blinded to performing sonographers. Quality and interpretability scores of novice- and expert-obtained LUS images were compared. Thirty volunteers with average age of 42.8 years (Standard Deviation (SD) 15.8), and average body mass index of 23.7 (SD 3.1) were recruited. Quality of novice and expert scans did not differ (median score 2.6, interquartile range (IQR) 2.3–2.9 vs. 2.8, IQR 2.3–3.0, respectively p = 0.09). Individual zone quality also did not differ (P > 0.05). Interpretability of LUS was similar between expert and novice scanners (median 7 zones interpretable, IQR 6–8, for both groups, p = 0.42). Interpretability of novice-obtained scans did not differ from expert scans (median 7 out of 8 zones, IQR 6–8, p = 0.42). Novice-users can self-obtain interpretable, expert-quality LUS clips with minimal training. Patient-performed LUS may be feasible for outpatient home monitoring.

## Introduction

Since early 2020, the Coronavirus disease 2019 (COVID-19) pandemic has placed unparalleled stress on healthcare resources worldwide. In response, and in conjunction with support from both private payers and government entities such as the Centers for Medicare and Medicaid Services, home-based health services and telemonitoring systems are rapidly expanding^1^. By mid-2021, telehealth use had surged to 38-fold higher than pre-pandemic levels, and a further 29% increase in telehealth visits for medical evaluation and management is anticipated by 2029^2,3^. As home-based initiatives evolve into a pillar of healthcare delivery, portable tools to facilitate diagnostics and therapeutics in alternative care settings such as patient homes must be explored.

Point-of-care ultrasound (POCUS) is a low-cost, radiation-free imaging modality that offers real-time data to aid medical care. Lung ultrasound (LUS) in particular demonstrates higher sensitivity than chest x-ray or computed tomography in detecting pulmonary pathologies such as pleural effusion, alveolar interstitial syndrome, pneumothorax, pneumonia, and acute decompensated heart failure^4–7^. For patients with heart failure, LUS findings over the course of treatment can predict adverse events and can correlate with both short- and long-term outcomes as well as patient mortality^8–11^. Expert consensus statements outlining the use of LUS to monitor known or suspected COVID-19 infection have also been proposed^12,13^. Over the last decade, POCUS has become even more portable with the development of hand-held transducers that collect and transmit images from smartphones or tablet devices. The versatility and increasing portability of POCUS makes this an ideal technology to deploy in alternative care settings.

The potential for patients to self-perform POCUS scans and obtain interpretable images to aid in real-time medical decision-making could transform home-hospital care. Traditionally POCUS experts undergo extensive training to learn to accurately acquire and interpret images^14^. Recent case reports have described healthcare providers who are infected with COVID-19 and on home isolation successfully performing daily self LUS to monitor disease progression^15,16^. However, these patients did have prior POCUS training, thus the ability for a typical home hospital patient with no prior POCUS experience to perform and obtain interpretable images remains unclear.

### Aim of the study

The goal of this work was to assess the ability of novice POCUS users to self-acquire interpretable LUS images using a hand-held device. We hypothesized that with minimal training, novice users would be able to self-obtain LUS images that were sufficient for diagnostic purposes, and which would have minor variation from the quality of expert-obtained scans.

## Methods

### Study design and setting

This prospective study was performed in participant homes outside of the healthcare setting. All patients provided informed consent for study participation and to publish their information. This work was performed in accordance with all local guidelines and regulations and was approved by the Mass General Brigham Institutional Review Board.

### Selection of participants

Healthy adult volunteers aged 18 years or older with no prior medical training were recruited. Individuals who met these criteria were referred to study personnel by colleagues and were recruited via telephone contact. Participants were deemed eligible for inclusion if they had access to a computer, email address, and internet to preview a slide show-based presentation which was electronically sent to them ahead of the study period, and were physically able to hold a probe on their chest as self-determined by participants. Live and/or continuous internet access during the study period was not required. Patients with prior medical or imaging training or prior experience performing POCUS were excluded from this study. Patients provided demographic information including age, height, weight, and highest level of formal education achieved.

### Novice training and LUS scanning protocol

A 28-slide tutorial slideshow with instructions on how to perform self-ultrasound was electronically mailed to participants to review on their own before initiating the study. The slideshow consisted of a combination of text and images including an introduction to POCUS, depicting use of the portable POCUS device and software, as well as desired LUS scanning technique and locations. Key anatomical structures necessary for obtaining interpretable images including rib shadows, pleural line, and A-lines as illustrated by the BLUE protocol and the American College of Emergency Physicians Emergency Ultrasound imaging criteria were described and depicted in the tutorial slideshow^17,18^. Training slides did not include information about pulmonary pathology identifiable on POCUS as the purposes of this study was to assess image acquisition alone and not image interpretation. Participants were allowed to review the slide show as many times as they needed to feel comfortable with the content prior to scanning, and were allowed to review the slides while scanning as needed. Study personnel did not assist or participate in patient-performed scans.

**Figure 1:**
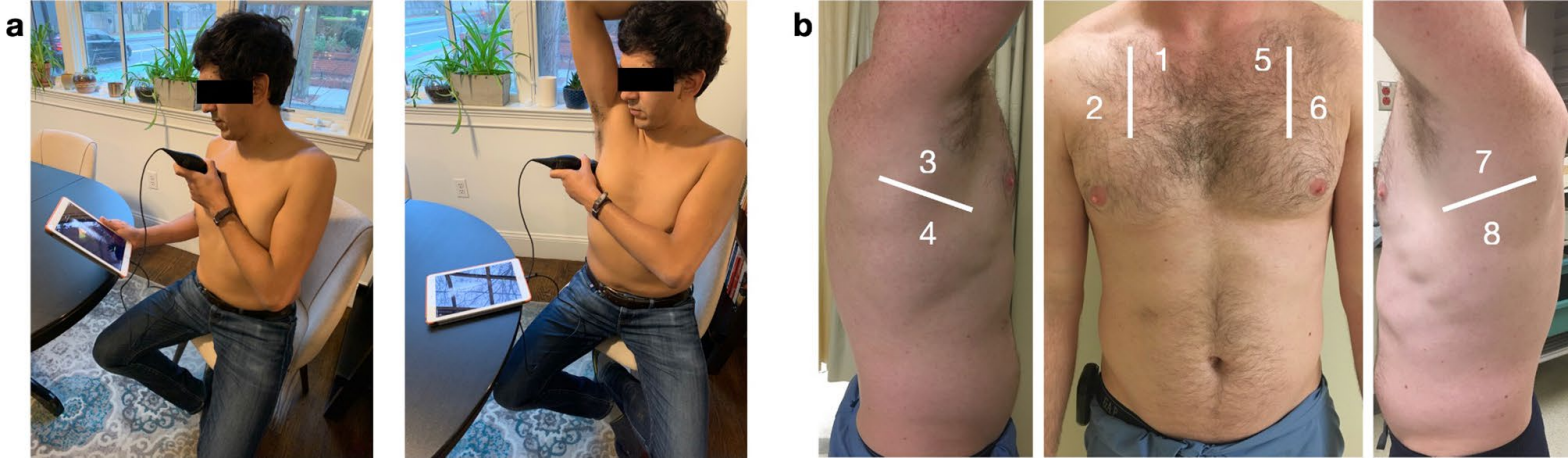
Novice-performed lung ultrasound scanning protocol. (**A**) A novice self-performs a LUS using a hand-held ultrasound probe attached to an iOS device. (**B**) 8-zone scanning protocol performed by both novices- and expert-sonographers.

LUS scans were performed in participant homes using a hand-held ultrasound probe (Butterfly iQ, Butterfly Network Inc, Guilford CT, USA) connected to an iOS-capable device (Apple, Cupertino CA, USA). After reviewing the tutorial, participants were asked to perform an 8-zone LUS on themselves with instruction to try to obtain as interpretable images as possible as described in the tutorial (Fig. 1A). Lung zones included two anterior zones and two lateral zones on each side of the thorax (Fig. 1B). While international standards for performing LUS have recently been proposed using 14-zone scans, these scanning protocols include 6 posterior lung zones which are not accessible to individuals performing self-scans, thus an 8-zone scanning protocol was used^13^. Participants were instructed to obtain the most interpretable clips possible for each zone following interpretability rules as described in the training slideshow. POCUS experts observed all novice-scans but did not offer instruction or intervene in the scanning in any way. Emergency medicine faculty with fellowship training in emergency ultrasound then repeated the same 8-zone LUS on each participant using the same ultrasound device. Six-second clips of each zone for both novice and expert scans were saved to the iOS device and subsequently uploaded to the cloud.

### LUS scoring and rater training

All LUS clips were deidentified and presented in random order to two POCUS experts (emergency medicine faculty with fellowship training in emergency POCUS, NMD and AJG). All images were blinded and randomized as to who acquired each clip (novice vs expert), and to lung zone scanned.

**Figure 2:**
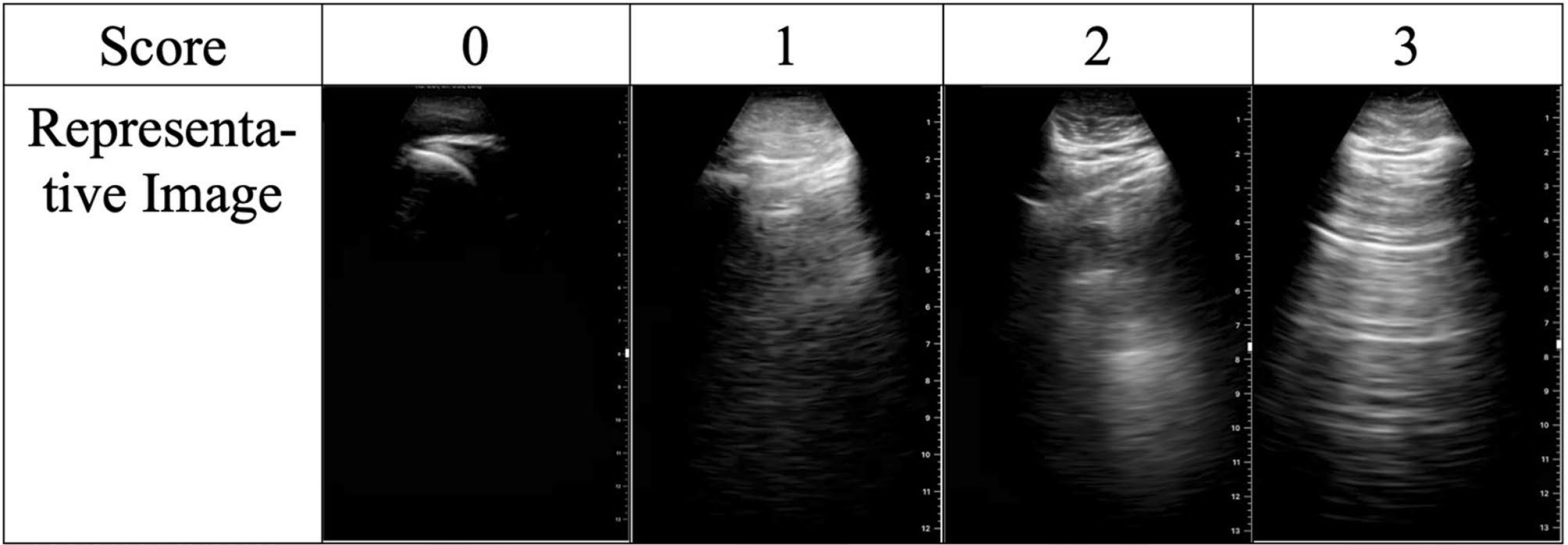
Scoring system used for grading novice- and expert-performed lung ultrasounds.

Clips were individually scored by each POCUS expert using a previously described scoring system as follows: 0 indicates no identifiable structures are visualized over an entirety of the clip, 1 indicates the pleural line is partially visualized but the clip is of non-diagnostic quality, 2 indicates that the pleural line is partially visualized but the image is sufficient for diagnostic purposes, and 3 indicates an ideal image with the pleural line easily visualized^15^. Scores of 0 and 1 were considered non-diagnostic whereas scores of 2 and 3 were considered to be sufficient quality for diagnostic assessment (e.g. interpretable, Fig. 2).

Prior to scoring study clips, for training purposes, two expert raters scored together a separate training dataset of LUS clips, discussing and resolving any rating discrepancies. The two raters then scored an additional dataset of 16 LUS clips independently to ensure acceptable inter-rater reliability. Post-training inter-rater reliability of quality scores was excellent (intraclass correlation coefficient 0.97, 95% CI 0.90–0.99). Agreement of image interpretability between the two raters was moderate (kappa = 0.64), with only one disagreement in interpretability. This disagreement occurred on a clip that was given a rating of 1 (pleural line is partially visualized in the clip but the clip is non-diagnostic in quality) by one expert, and a score of 2 (pleural line is partially visualized in the clip however the clip is diagnostic in quality) by the second expert.

### Statistical analyses

Data were analyzed using standard parametric and non-parametric techniques. The number of interpretable zones and quality scores of novice-obtained scans were compared with expert-obtained scans using Wilcoxon signed rank sum tests. Proportions of interpretable scans between groups were compared using Chi-square tests. To account for multiple comparisons, we used Bonferroni-adjustments. Agreement in ratings between the two trained raters on quality scores and interpretability of scans was assessed using intraclass correlation coefficients and Kappa statistics, respectively. All analyses were performed using SAS version 9.4 (SAS Institute Inc., Cary, NC, USA) and SPSS, version 28 (IBM Corporation, Armonk, NY, USA).

## Results

### Characteristics of study subjects

In total, 30 participants were recruited for this study. Participants had an average age of 42.8 years [standard deviation (SD) 15.8], an average body mass index of 23.7 kg/m^2^ (SD 3.1), and 67% of participants (n = 20) were male (Table 1). Among participants 3% were high school graduates (n = 1), 3% received some college credit but no degree (n = 1), 27% held a Bachelor’s degree (n = 8), 23% held a Master’s degree (n = 23), 13% held a Professional degree, and 13% held a Doctoral degree (n = 9).

**Table 1:** Participant demographics.

Characteristic	Subjects, N = 30 [no. (%)]
**Sex**	
Male	20 (67)
Female	10 (33)
**Age (years)**	
20–30	9 (30)
31–40	7 (23)
41–50	4 (13)
51–60	2 (7)
61–70	8 (27)
**Ethnicity**	
Hispanic or Latino	6 (20)
Not Hispanic or Latino	24 (80)
**Race**	
American Indian or Alaskan Native	0 (0)
Asian	3 (10)
Native Hawaiian or Pacific Islander	0
Black or African American	1 (3)
Caucasian	18 (60)
Other	8 (27)
**Body Mass Index (kg/m**^2^**)**	
< 18.5	1 (3)
18.5–24.9	22 (73)
25–29.9	6 (20)
30–39.9	1 (3)
> 40	0 (0)
**Educational background**	
No schooling completed	0 (0)
Some highschool, no diploma	0 (0)
High school graduate, diploma or equivalent	1 (3)
Some college credit, no degree	1 (3)
Trade/technical/vocational training	0 (0)
Associate degree	0 (0)
Bachelor’s degree	8 (27)
Master’s degree	7 (23)
Professional degree	4 (13)
Doctorate degree	9 (30)

### Main results

A total of 480 LUS clips were obtained from both novice and expert POCUS scanners. Overall, 84% (n = 201) of novice-obtained clips were considered interpretable, which was not different compared to 87% expert-obtained clips being interpretable (n = 209), p = 0.30. Quality scores of novice and expert scans did not differ (median 2.6, interquartile range (IQR) 2.3–2.9 for novices, median 2.8, IQR 2.3–3.0, p = 0.09 for experts) (Table 2). Quality scores in the eight individual zones also did not differ (P > 0.05 for all). Overall, a median of 7 out of 8 zones were deemed interpretable for novice-obtained scans (IQR 6–8). Interpretability of novice- and expert-obtained scans did not differ, compared to expert-obtained scans (median 7 out of 8 zones, IQR 6–8, p = 0.42). When adjusted for multiple comparisons, interpretability of LUS for each zone did not differ between expert-obtained scans and novice-obtained scans (Table 2).

**Table 2:** Novice- vs expert-obtained clip quality score reported at median average scores (interquartile range).

Zone	Novice score*	Expert score*	P-value**	Novice interpretable N (out of 30)	Expert interpretable N (out of 30)	P-value
1	3 (2–3)	3 (1–3)	0.63	23	21	0.01
2	3 (3–3)	3 (3–3)	1.00	26	28	1.00
3	3 (1–3)	3 (2–3)	0.18	21	24	0.005
4	3 (3–3)	3 (3–3)	0.75	27	29	1.00
5	3 (2.5–3.0)	3 (2.5–3.0)	0.75	26	24	0.02
6	3 (2.5–3.0)	3 (3–3)	0.23	26	28	1.00
7	3 (2–3)	3 (3–3)	0.39	27	27	0.02
8	3 (3–3)	3 (3–3)	0.13	25	28	0.31

*This score references the gold standard score which is the average of the expert raters’ scores.

**Wilcoxon signed rank sum test.

Inter-rater reliability between the two raters was high. Agreement was excellent for quality scores between the two raters (ICC 0.99, 95% 0.97–0.99 for both novice and expert-obtained scans) (Table 3). Agreement was also almost perfect for both novice and expert-obtained scans in the determination of interpretability [kappa for novice scans = 1.00 (CI 1.00–1.00); kappa for expert scans = 0.98 (CI 0.94–1.00)] (Table 3).

**Table 3:** Inter-rater reliability for scores of novice- and expert-obtained clips.

Zone	Scanner level	ICC	Kappa (CI)
1	Novice	0.97 (0.94–0.99)	1.00 (1.00–1.00)
1	Expert	1.00	1.00 (1.00–1.00)
2	Novice	0.96 (0.92–0.98)	1.00 (1.00–1.00)
2	Expert	0.88 (0.75–0.94)	1.00 (1.00–1.00)
3	Novice	0.98 (0.95–0.99)	1.00 (1.00–1.00)
3	Expert	0.99 (0.98–1.00)	0.89 (0.68–1.00)
4	Novice	0.96 (0.92–0.98)	1.00 (1.00–1.00)
4	Expert	0.96 (0.91–0.98)	1.00 (1.00–1.00)
5	Novice	0.96 (0.92–0.98)	1.00 (1.00–1.00)
5	Expert	0.99 (0.97–0.99)	1.00 (1.00–1.00)
6	Novice	0.95 (0.89–0.98)	1.00 (1.00–1.00)
6	Expert	0.99 (0.97–0.99)	1.00 (1.00–1.00)
7	Novice	0.96 (0.92–0.98)	1.00 (1.00–1.00)
7	Expert	0.96 (0.91–0.98)	1.00 (1.00–1.00)
8	Novice	1.00	1.00 (1.00–1.00)
8	Expert	0.97 (0.93–0.99)	1.00 (1.00–1.00)
Avg	Novice	0.99 (0.97–0.99)	All 8: 1.00 (1.00–1.00)
Avg	Expert	0.99 (0.97–0.99)	All 8: 0.98 (0.94–1.00)

Kappa values are reported for comparing clips rated as interpretable.

*CI* confidence interval.

## Discussion

In this study we assessed whether healthy volunteers with no prior medical experience or POCUS training could accurately perform LUS using a hand-held POCUS in their home setting. Overall, quality and diagnostic capabilities of LUS scans did not differ between novice- and expert-obtained scans. This work supports the emerging body of literature which suggests that non-physician novice POCUS users are able to self-perform LUS with minimal prior training and without the need for live expert guidance^11,15,19^.

Our data demonstrate that novice POCUS users can obtain LUS images of both anterior and lateral lung zones independently without need for expert assistance. This work supports the recent report by Kirkpatrick et al. in which novice users guided by experts on live tele-ultrasound were able to self-obtain images of the anterior and lateral lung zones in 100% of attempts with 99.8% of images being read as interpretable by experts^20^. However in this prior work only 66% of attempts to self-obtain images of posterior lung zones were successful. Our goal with this work was to create a LUS scanning protocol which is easy and feasible for novice users to successfully self-perform at home. However, for some pathological processes, important findings may be missed by not imaging posterior fields. Further work is needed to assess whether interpretable posterior lung zone clips are obtainable in remote patient self-assessment.

In recent years, healthcare initiatives focusing on expanding home-based care services have been rapidly expanding world-wide^1–3^. These initiatives which explore a variety of services such as monitoring post-hospital discharge, providing primary hospital services, and outpatient telehealth visits all provided remotely to patients in their own homes have shown promise in both clinical outcomes and patient satisfaction^21–25^. With the entry of portable, hand-held POCUS devices into the market, availability of POCUS for use outside of traditional acute care settings is now feasible. This includes settings where point-of-care imagine was not historically available such as in primary care offices, rural health clinics, and even in patient homes. As home-based healthcare services become more widely available, advanced methods for patient evaluation and monitoring must be explored.

**Figure 3:**
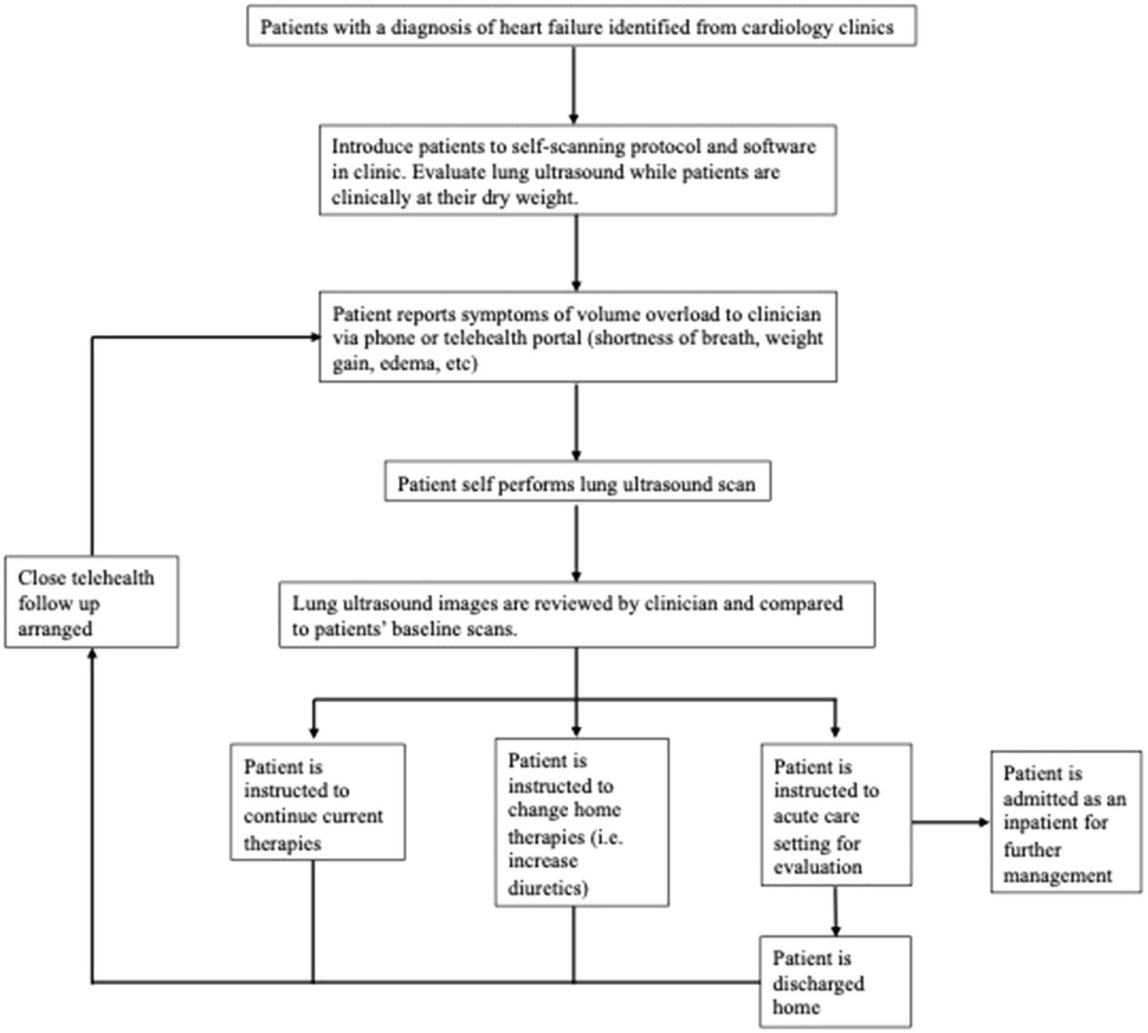
Proposed workflow for integrating patient-performed self-lung ultrasound into clinical care.

This work helps to demonstrate that POCUS may be a viable approach to patient-centered monitoring and diagnostic evaluation for conditions which are increasingly being managed outside of traditional clinical spaces such as heart failure. Currently, patients with heart failure are asked to monitor their weight and blood pressure at home daily to help guide outpatient medical management. LUS also has a demonstrated role in assessing acute heart failure exacerbations and the presence of B-lines on LUS has been correlated with increased morbidity and mortality in this population^9,10^. Thus, programs which incorporate tools such as LUS into home-based monitoring programs may improve disease management by providing more objective data for monitoring purposes. A suggested workflow to incorporate LUS into home-based monitoring for conditions such as heart failure can be seen in Fig. 3. While performing self-scans may be challenging in acutely ill patients, this may represent a viable tool for home monitoring patients with chronic illness or acute-on-chronic disease. Future studies examining clinical outcomes of such a program would provide invaluable information both for clinicians and private companies examining new technology related to remote or home-based healthcare.

## Limitations

This was a small, single center study which limits the generalizability of our findings. Subjects were recruited via referral from colleagues to study personnel, thus this may have led to selection bias in our study population. Our population had a fairly high level of baseline education which may have impacted their comfort with using and adjusting to new technology. Further, the average BMI of our participants was fairly low compared to the national averages of 29.5 for adult males and 30.1 for adult females^26^. Patients with a low BMI may have more flexibility in reaching each lung zone, or have less soft tissue to scan through thereby improving ease of obtaining interpretable images^27^.

Subjects enrolled in the study were assessed only once in their LUS self-exam and as a result we did not assess their ability to reliably perform the exam over time. As prior studies have shown that patients can reliably perform repeat self-medical evaluations such as blood pressure and/or blood glucose at home reliably, further longitudinal studies are needed to determine whether patients can reliably perform self- LUS and obtain interpretable scans over time.

## Conclusions

As healthcare increasingly evolves towards providing care in alternative settings such as virtual telehealth visits or in patients’ homes with home hospital programs, portable tools that offer rapid, accurate data and can be self-implemented by patients will be needed. Here, we demonstrate that LUS when performed by healthy volunteers is not only feasible, but provides interpretable and diagnostic images. The applicability of these images to guiding clinical decision-making in home-based care should be explored.

## Supplementary Information

Supplementary Information.

## Data Availability

The datasets during and/or analyzed during the current study are available from the corresponding author on reasonable request.

## Abbreviations

BMI: Body Mass Index
COVID-19: Coronavirus disease 2019
LUS: Lung ultrasound
POCUS: Point-of-care ultrasound
IQR: Interquartile range
SD: Standard deviation
